# Oncolytic herpes simplex virus and immunotherapy

**DOI:** 10.1186/s12865-018-0281-9

**Published:** 2018-12-18

**Authors:** Wenqing Ma, Hongbin He, Hongmei Wang

**Affiliations:** Ruminant Diseases Research Center, Shandong Provincial Key Laboratory of Animal Resistance Biology, College of Life Sciences, Shandong Normal University, Jinan, 250014 China

**Keywords:** Oncolytic herpes simplex virus, Cancer, Immune escape, Genetically engineered, Oncolytic viral therapy

## Abstract

**Background:**

Oncolytic viruses have been proposed to be employed as a potential treatment of cancer. Well targeted, they will serve the purpose of cracking tumor cells without causing damage to normal cells. In this category of oncolytic viral drugs human pathogens herpes simplex virus (HSV) is especially suitable for the cause. Although most viral infection causes antiviral reaction in the host, HSV has multiple mechanisms to evade those responses. Powerful anti-tumor effect can thus be achieved via genetic manipulation of the HSV genes involved in this evading mechanism, namely deletions or mutations that adapt its function towards a tumor microenvironment. Currently, oncolytic HSV (oHSV) is widely use in clinical; moreover, there’s hope that its curative effect will be further enhanced through the combination of oHSV with both traditional and emerging therapeutics.

**Results:**

In this review, we provide a summary of the HSV host antiviral response evasion mechanism, HSV expresses immune evasion genes such as ICP34.5, ICP0, Us3, which are involved in inducing and activating host responses, so that the virus can evade the immune system and establish effective long-term latent infection; we outlined details of the oHSV strains generated by removing genes critical to viral replication such as ICP34.5, ICP0, and inserting therapeutic genes such as *LacZ*, granulocyte macrophage colony-stimulating factor (GM-CSF); security and limitation of some oHSV such G207, 1716, OncoVEX, NV1020, HF10, G47 in clinical application; and the achievements of oHSV combined with immunotherapy and chemotherapy.

**Conclusion:**

We reviewed the immunotherapy mechanism of the oHSV and provided a series of cases. We also pointed out that an in-depth study of the application of oHSV in cancer treatment will potentially benefits cancer patients more.

## Review

### Introduction

For the past few years,despite constant new attempts finding phenomenal cancer treatments, chemotherapy, radiation and targeted drugs therapy are still the main therapeutic method in clinical practice. However, many problems remain in these methods, such as incompleteness, severe side effects, easy development of drug resistance, and lack of control in tumor recurrence and metastasis, etc., all of which lead to unsatisfactory result in treating tumor. The shortcomings of these major therapies call for new strategies in the field of cancer [1].

The Oncolytic virus is a subtype of a lytic virus that selectively replicates and kills cancer cells and spreads within the tumor without damaging normal tissue. The activities of oncolytic virus reflect the basic biological principles of the virus and the interaction of host-virus in the fight between pathogenesis and the immune system [2].

HSV, a member of the alpha-herpesviruses subfamily, shares many similarities with pseudorabies virus, varicella-zoster virus and infectious bovine rhinotracheitis virus [3]. The virus contains double stranded DNA genomes of at least 120 kb, encoding for 70 or more genes. At present, lysotype HSV is the first virus to be developed into a recombinant oncolytic viral therapeutic vector, and the first oncolytic virus to fight cancer. As a cytolytic virus HSV possesses the following advantages: (1) HSV replicate quickly in cells and has capability to infect multiple types of cancer cells; (2) HSV has a large genome, which can be easily modified and be inserted with multiple additional transgenes [4, 5]; (3) HSV can be prevented with antiviral drugs when the dose start to impose threat to the patients’ lives [6–8]; (4) Modifying the glycoprotein of HSV can improve the targeting of tumor cells [9].

**Fig. 1:**
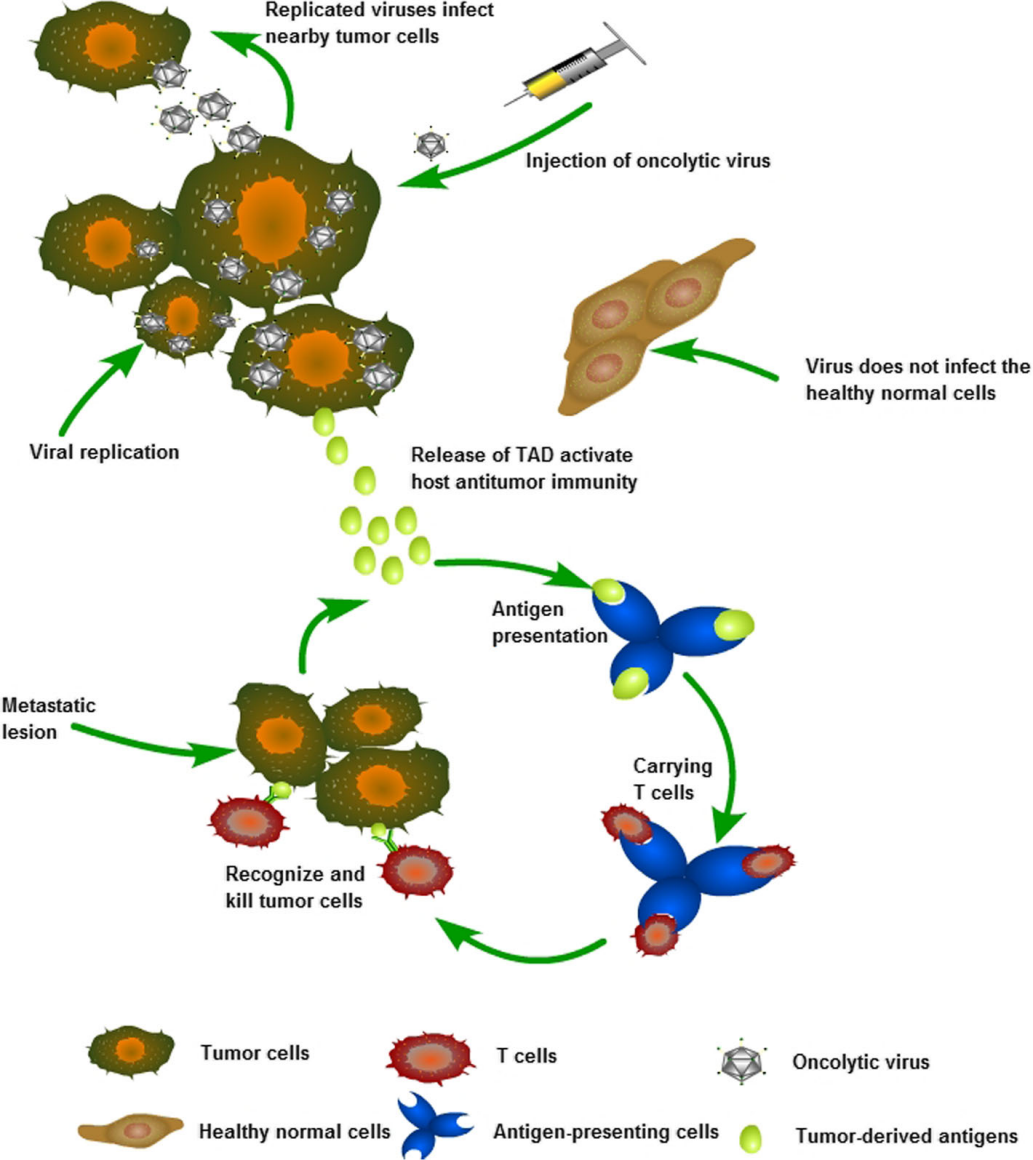
Mechanisms of oncolytic virus selective killing tumor cells. Local replication of oncolytic virus induces lysis of tumor cells results in release of tumor-derived antigens which promote the activity of the cancer-immunity cycle, resulting in the specific antitumor immunity in the course of its oncolytic activities that act on remote lesions, ultimately killing the tumor cells selectively

As efficient OVs, HSV has some ability to escape the host’s immune response including: To complement and incapacitate immunoglobulins via viral glycoproteins; to inhibit the production of cytokine/chemokine from infected cells [10]; to block the antigen presenting cells’ (APCs) maturation [11]; to evade host immunological surveillance via negative-regulation of the expression of MHC class I [12] and to inhibit the apoptosis and cell death induced by cytotoxic T lymphocyte(TL) [13]. For deletion or mutation of those genes that were involved in HSV’s escape through its host’ immune defense will prohibit its replication in normal cells. Tumor microenvironment is often in an immunosuppressive state, which may allow the virus’ entry and replication, which in turns eventually leads to the dissolution and death of tumor cells (Fig. 1). oHSV can also reverse the immune suppression of tumor microenvironment, enhance tumor immunogenicity, promote the infiltration of inflammatory cells, and play an effective anti-tumor effect.

This review elaborates on how the HSV surmounts the anti-viral defense mechanism of the host; the oHSVs’ involvements in deletion or modification of viral gene and the clinical development of oHSV. A better understanding of the complex pattern of the interaction between HSV and host, and combination with current clinical oHSV is essential to the refinements the strategy of oHSV, thus to improve the therapeutic effects and to comprehend the oHSV immune imperfection.

### The mechanism of HSV confronted the host immune response

**Fig. 2:**
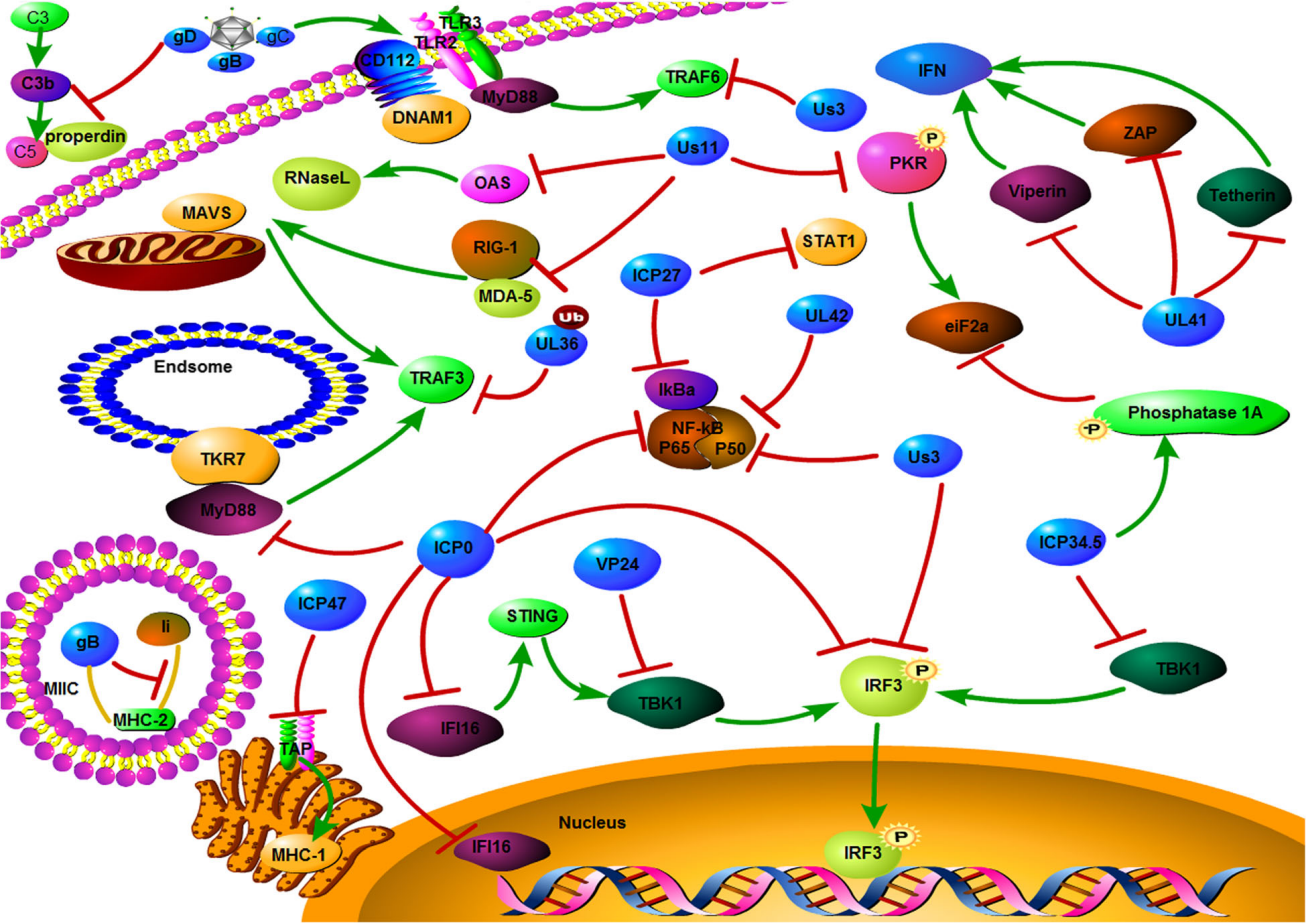
Mechanism of HSV evades host immune responses. HSV expresses genes which evade host immune surveillance via inactive immune regulation factor involved in the antiviral inmate immunity pathway, such the TLR signaling pathway, RLR signaling pathway and the DNA sensor signaling pathway

The infection of HSV causes to a cascade reaction of host anti-viral immunity responses. As a successful pathogen, HSV expresses proteins which are involved in inducing and activating host responses, so that the virus can escape from the immune system and set up effective long-term latent infection. HSV has various mechanisms to escape the host reactions (Fig. 2):

### Envelope glycoprotein

The envelope glycoproteins of HSV can escape humoral immunity mechanisms, such as Glycoprotein C binding and inactivating properdin and complement protein C3b, C5 to protect the virus from virus neutralization mediated by complement induced via natural IgM and antibody-independent complement neutralization [14, 15]. gE and gI encode Fc receptors, which can attach to IgG [16]. This binding inactivation of complement mediated via antibody and cytotoxicity of antibody dependence conduce pathogenicity [17]. gD inhibit the expression of CD112, which binds to the natural killer (NK) cells excitating receptors DNAX accessory molecule 1 (DNAM1), leading to a noneffective binding and lysis to HSV-infected or gD-transfected cells via NK [18]. HSV inhibits the expression of CD1d, surface molecules of APCs, and thus reduces NKT cells stimulate [19].

### Immune evasion genes

A series of genes encoded via HSV escape the host antiviral immune monitoring mechanism [10, 20]. In cancer cells, some of these pathways are flawed [21]. Interferon (IFN) 1 signaling pathway, crucial for antiviral innate immunity [22], relates to genes that are involved in pathways such as the TLR signaling pathway: Us3 inhibit the signal transmission of TLR3 and TLR2 to TRAF6 [23], deubiquitinase for UL36 down-regulate the expression of TRAF3 [20], ICP0 inhibite the expression of P50 and P65, the subunits of MyD88 and NF-κB [24], UL42 and Us3 inhibit the process of nuclear translocation via NF-κB [25, 26], and ICP27 bind to IκBa inhibits NF-κB [27]. In the RLR signaling pathway: the Us11 can combine with RIG-I and MDA-5, thus inhibits the integration with MAVS [28, 29]. ICP34.5 inhibit the phosphorylation of IRF3 by binding TBK1 [30], US3 inhibit production of IFN-β via hyperphosphorylation of IFN regulatory factor 3 (IRF3) [31], ICP0 prevents IRF3 sustained activation, thus inhibiting translocation from the nucleus to cytoplasm [32]. Influence of the DNA sensor signaling pathway is also present: IFI16, involved in sensing pathogen DNA and initiating signaling pathways, inhibits via ICP0 in the nucleus [33]. In addition, IFN-stimulated genes, UL41 the virion host shutoff protein (Vhs), inhibits viperin, ZAP, and tetherin via degraded mRNA [20, 34], Us11 inhibits OAS [35] and protein kinase R (PKR) [36]. Eukaryotic Initiation Factor 2 (eIF2a) phosphorylated via PKR, shuts down the synthesis of protein. ICP27 blocks the phosphorylation and activation of STAT-1 [37]. Due to the defection of cancer cells in IFN signaling [38], deletion or mutation in these immune evasion genes such as ICP34.5, ICP0, Us3 in oHSV will avail ourselves of a cancer therapy [21].

### Block dendritic cells function

Dendritic cells (DCs) are polymorphisms and heterogeneous antigen presenting cells, it has vital function for the recognition of pathogens at the site of infection and the initiation of protective HSV-specific T cells [39]. HSV has numerous mechanisms to inhibit DCs function [11]. ICP34.5 binds to TBK1 and IKKα/β, impeding the maturation of DCs and inhibits autophagy by interfering with antigen presentation [40, 41]. ICP0 induces CD83 degradation as a DCs maturation marker, leading to a decrease in T cell stimulation [42] ICP47 blocks transporter associated with antigen presentation (TAP), inhibits MHC I-peptide presentation, and thus leads to a mediated by MHC I to CD8+ T-cells to escapes immunological surveillance in host cells without antigen presentation. Pourchet A et al. showed that oHSV express UL49.5 from BHV-1 has a high efficacy treating cancer models, which is rely on CD8+ T cells [43]. gB binds to HLA-DM and HLA-DR, which yields a negtive-regulation of MHC II pathway in CD4+ T cells [44]. ICP34.5 and UL41 interfere with antigen presentation of CD4+ T cells via down-regulated MHC II accumulation on the surface of glioblastoma cells [45].

T cells are also influenced via HSV infection. Firstly Us3 inhibits LAT, which is the linker activating T cells, and in turns blocks TCR signaling [24]. Secondly oHSV infection has an impact on the pathway in T cells, such as inhibiting NF-κB, activating STAT3, JNK and MAPK p38 pathways, and suppressing the pro-inflammatory cytokines synthesis, such as IL-2, TNF-a and increasing IL-10 synthesis [46].

### Inhibition of autophagy

Autophagy is an important cellular degradative pathway [47], which exerts on cellular pathogens like oHSV with a process similar to the MHC I and II presented in APCs [48]. ICP34.5 targets Beclin1 and interacts with PPP1CA, blocking the formation of autophagosome [49–51]. HSV induced EIF2AK2 activation down-regulates the Beclin1-mediated autophagy [49, 51]. In additional, ICP34.5 directly inhibits TBK1, which can regulated the phosphorylation of autophagic receptors SQSTM1/p62 and optineurin (OPTN) to mediate substances recruitment into phagophores for degradation [52]. Us11 interacts with EIF2AK2, inhibiting the phosphorylation of EIF2S1, mediated via EIF2AK2, to block autophagy [53].

### Inhibition of apoptosis

Apoptosis, the programmed cell death, can clear up the infected cells. HSV encoding anti-apoptotic virulence factors to suppress apoptosis then gives the virus enough time to replicate after infection [13, 54]. After HSV infection, some genes like ICP6, Us3, gD and Us5 (gJ) play role in the suppression of cells apoptosis. Us3 suppresses the expression of cytochrome c and the activation of caspase-3 [54], Us3 protein kinase activates the proapoptotic proteins Bad and Bid [55]. Us5 (gJ) antagonizes Fas/UV-induced apoptosis and weakens the granzyme B-mediated pathways of CTL-induced apoptosis [56]. Compared to gJ, Us6 (gD) blocks apoptosis at different stages of the viral life cycle. Necroptosis, another programmed cell death, which absence of caspases. ICP6 suppress apoptosis by blocking caspase 8 mediated via TNF-α and Fas ligand [57], also blocking necroptosis induced by TNF via inhibits the binding of RIP1 and RIP3 [58]. However, ICP6 had the reverse effect, in mice [13, 59].

The interactions of these host viruses are crucial to harmonize the OV activity, regulate the OVs anti-viral immune responses, and inducing anti-tumor immunity.

### Genetically engineered oHSV

The key to eradicate tumors in this new therapy is to improve the precision when targeting oHSV for tumors, and enhance the suppression. Genetic engineering can affect many aspects of how viruses’ work. To enhance tumor selectivity by removing key genes in healthy cells that replicate with the virus. Table 1 summarizes the modified viral genes in oHSV and the functions of viral proteins encoded by these genes.

**Table 1:** Immune evasion genes of HSV

Gene	Protein	Function	oHSV name
UL27	gB	Part of initial attachment of the virus to the cell by binding to heparan sulfate. With gH/gL, enables fusion of the envelope with the cell membrane. Down-regulation of MHC II processing pathway in CD4+ cells.	R5141; KNE
UL44	gC	Forms the initial attachment of the virus to the cell by binding to heparan sulfate. Inactivates serum complement proteins.	R5141
US6	gD	Binds to HVeM and/or nectin-1, leading to a conformation change that initiates fusion. Down-regulates NK receptor ligand and NK-mediated lysis; inhibition of apoptosis.	R5141; R-LM249; HSV1716EGFR; KNE
RL1	ICP34.5	Major neurovirulence gene. Suppression of PKR/eIF-2a signaling pathway and IFN-induced anti-viral mechanisms; Inhibits DC maturation and antigen. Presentation; Blocks MHC class II accumulation on the cell surface; Binds to Beclin-1, inhibiting autophagy.	HSV1716; R3616; OncoVex^GMCSF^; G47; ΔG207; DM33;
RL2	ICP0	Blocks NF-κB-mediated transcription of immunomodulatory cytokines, and IRF3-induced and IRF7-induced anti-viral signaling pathways; inhibits IRF3 translocation to the nucleus; inhibits IFI16; degradation of mature DC marker (CD83). Involved in transcription of viral genes. Has ubiquitin ligase activity. Inhibits interferon response. Alters the cellular environment to promote viral replication.	R7020 (NV1020);
UL39	ICP6	Major subunit of ribonucleotide reductase. Blocks TNF-a-mediated and Fas ligand-mediated apoptosis through interacting with caspase 8 and necroptosis.	hrR3; G47Δ
UL54	ICP27	Inhibits cellular mRNA splicing. Recruits necessary proteins involved in viral transcription and translation. Activates cellular pathways to promote viral replication. Blocks NF-κB and IRF3 signaling pathways; blocks STAT1 activation and its translocation to the nucleus.	HF10
US12	ICP47	Down-regulates MHC class I by inhibiting TAP.	G47Δ; OncoVex^GMCSF^
US11	US11	Binds to and is phosphorylated by PKR, preventing cellular inhibition of protein synthesis and autophagy; blocks OAS.	G47Δ
US3	US3	Inhibits NF-κB activation and reduces cytokine expression, such as IL-8; inhibits induction of apoptosis; hyperphosphorylates IRF3 to block activation of RLR signaling pathway.	R7041
UL48	VP16	Initiates transcription of immediate early genes. Inhibits NF-κB activation and blocks IRF3 pathway and IFN-β production.	KM100

Dlsptk, the first type 1 herpes simplex virus mutants, includes a mutation within the UL23 gene that encodes the thymidine kinase (TK) gene. Dlsptk can inhibit the growth of glioma in nude rat brain [60, 61]. Nevertheless, high doses of Dlsptk can cause fatal encephalitis. For this reason, it is necessary to look for other engineered herpes mutants with low toxicity [62].

HrR3, the recombinant HSV-1, insert a *LacZ* in HSV-1 UL39 (encode ICP6), alternatively replicates in cancer cells, which has remarkable anticancer activity [63, 64].

HSV1716 was isolated from HSV-1 (17+) strain, deletes two copies of the main neurotoxic determinant generepeat RL1, which encodes neurotoxic determinant ICP34.5). PKR phosphorylates eIF2a, thus inhibits protein translation and induces cell apoptosis and kills the virus. ICP34.5 mediated dephosphorylation of eIF2a prevent cell apoptosis and protect the survival and reproduction of virus [65]. 1716 targets cancer cells that uncontrollable protein synthesis [66].

R3616, isolated from HSV-1 (F) strain, deletes the two replicas of ICP34.5 genes. R3616 can effectively induce host anti-tumor immune response by inducing a series of immune cells [67]. Kanzaki et al. showed that R3616 infects tumor antigen-specific lymphocytes; this not only effect on primary tumors, but also regulates multiple metastases [68].

NV1020 is an attenuated HSV that contains a diploid gene (RL1, RL2 and s1), with UL56 in the genome deleted [69]. Moreover, NV1020 attenuated via delete the TK gene and the UL24 genes promoter, and then inserts an exogenous copy of TK gene. These changes allow NV1020 highly attenuated and only proliferates in tumor cells.

G207, the first oHSV to be tested in clinical trials, deletes the ICP34.5 and inserted the *LacZ* gene, so the virus can selectively spread in tumor cells [70]. The deletion mutants ICP34.5 induced the down-regulate of late viral genes including US11 via PKR [9]. G207 can induce systemic anti-tumor immunity, which is related to the activation of cytotoxic T lymphocytes [8].

G47Δ derived from G207, contain two of the mutations in the RL1 and ICP47 genes, and insert the *LacZ* in ICP6 gene (coding ribonucleic acid reductase large subunit) area cause its inactivation. Inactivation of ICP6 then induces the oHSV’s only replicate in proliferating cells. Furthermore, the ICP47 mutation can effectively activate the host’s anti-tumor immune response via enhanced MHC-I expression [71]. Due to the three remoulds in the genome, G47Δ may be less toxic and more secure than G207 and T-Vec.

DM33 includes deletions of ICP34.5 and LAT gene. Unlike Dlsptk, DM33 was isolated from the McKrae strain, which promotes viral growth and kills cancer cells [72, 73].

HF10, remove the 3.9 kb connection point between the right end of UL and UL/IRL, which caused the loss expression of UL56, and reproduction of UL53 (gK), UL54 (ICP27) and UL55 [9]. HF10 enhances angiogenesis and induces acytotoxic T lymphocytes anti-tumor response [62].

Oncovex^GM-CSF^, ICP34.5 and ICP47 genes in HSV-1 were strike out, and the integration of human GM-CSF was step in the ICP34.5 site. A series of cytokines such as IL12, GM-CSF, IFN-α and tumor necrosis factor (TNF-α) used with oHSV can modify and enhance the anti-tumor immunity. The GM-CSF shows the most effective results. TNF-α, IL-12 and IFN-α preclinical cancer studies have also show promising contributions [74, 75]. Oncovex^GM-CSF^ enhanced antigen-specific T cell response and decreased inhibitory CD4+ regulation of T cell expression, with a specific antitumor effects achieved in CD8+ T cells [76].

### Clinical development and limitations of oHSV

Oncolytic viruses have assessment for treatment of a series of mlignation tumors. The first clinical trials of engineered virus was conducted in the 1990s [77]. Several different oHSV have been or will be tested worldwide for various cancers; some have been developed to phase II/III trials, such as G207, 1716, OncoVEX, NV1020, HF10, G47Δ (Table 2) [78].

**Table 2:** oHSVs of genetic engineering and its clinical application

oHSV name	Genetic modification	Descrption	Clinical application
Dlsptk	TK^−^(UL23)	Internal deletion within UL23	Malignant human gliomas
hrR3	UL39	Insertion of *LacZ* (encodes β-galactosidase) in UL39	Pancreatic cancer; colon carcinoma; liver cancers
HSV1716	ICP34.5	Deletion in both copies of ICP34.5	Glioblastoma multiforme; anaplastic astrocytoma; oral squamous cell carcinoma
R3616	ICP34.5	Deletion of two copies of ICP34.5	Pancreatic cancer; colon carcinoma
G207	ICP34.5	Deletions of two copies of the ICP34.5; insertion of an *Escherichia coli LacZ*	Prostate adenocarcinoma; glioblastoma; hepatocellular carcinoma; colorectal cancer
R7020 (NV1020)	UL23, UL55, UL56, RL1, RL2, RS1	Deletion of UL23, as well as the region encoding UL55, UL56, and one copy of RL1, RL2, and RS1 (though not the RS1 promoter)	Pancreatic cancer; colon carcinoma; bladder cancer; pleural cancer
G47Δ	RL1, UL39, US11, US12	Deletion of the overlapping US11 promoter/US12 region, putting expression of the normally late US11 gene under the immediate early US12 promoter	Prostate adenocarcinoma; glioblastoma; rectal cancer; nasopharyngeal carcinoma; breast cancer
OncoVex^GM-CSF^	ICP34.5 and ICP47	Deletion of two copies of ICP34.5 gene and the viral ICP47 genes; insertion of GM-CSF	Breast cancer; head and neck cancer; gastrointestinal cancers; malignant melanoma
HF10	UL53, UL54, UL55, UL56	Spontaneous deletion of UL56 as well as duplication of UL53, UL54, and UL55	Breast cancer; malignant melanoma; pancreatic cancer
DM33	ICP34.5	Deletions of γ-34.5 and LAT gene	Human gliomas and glioma cell line

Initially, oHSVs lay emphasis on security vectors, which included the deletes ICP34.5 gene, such as HSV1716. HSV1716 was first demonstrated to be safe and toxic in patients with pleomorphic glioblastoma and intersex astrocytoma [79]. The results showed that HSV1716 had good tolerance and no adverse reactions occurred after high dose of 1 × 10^5^ PFU treatment. HSV1716 has been used in the treatment of glioma and oral squamous cell carcinoma [80–82].

Then strains with additional multiple deletions or mutations in case of the reversion of wild type virus, like the G207 [83], became the first oHSVs used in clinical trial. G207 has been used for recurrent malignant glioma in phase I studies, untoward effect have been moderated to slight fever and local erythema/inflammation reactions at the sites of injection [84].

G47Δ enhances the anti-tumor efficacy while retaining the safety characteristics of G207 [85–87]. G47Δ showed efficacy in all solid tumor models tested in vivo, such as hepatocellular carcinoma [88], schwannoma [89], prostate cancer [87, 90, 91], nasopharyngeal carcinoma [71], glioma, thyroid carcinoma [92], colorectal cancer, breast cancer [93] and malignant peripheral nerve sheath tumor. G47Δ has the ability to killing cancer stem cells [94]. At present, G47 Δ is the only third generation oHSVs tested on humans [85].

NV1020 safety and efficacy have been demonstrated in some cancer diseases, such as colon carcinoma, pleural cancer, bladder cancer and pancreatic cancer [95–98]. Kemeny N et.al investigated the safety and tolerance of NV1020 in liver metastasis of colorectal cancer in a phase I clinical trial [99]. NV1020 was also tested for liver metastasis from colorectal cancer in phase II trials. The results show that NV1020 is safe and effective in anti-tumor therapy [100]. In phase III trials, NV1020 was used in combination with cytotoxic and targeted drugs [62].

Oncovex^GM-CSF^ is the first type of oncolytic virus. After genetic engineering, oncogm-csf can selectively replicate in tumor cells, directly inject into the lesion, express GM-CSF, and enhance systemic anti-tumor immune response [68, 101]. GM-CSF insertion can promote complementary anti-tumor immune response by recruiting APCs [102]. After intratumoral injection of oncovex^GM-CSF^, the lesions of 8/50 patients with metastatic malignant melanoma disappeared completely [103]. The safety of an oncovex^GM-CSF^ has been determined in phase I studies [104]. Direct injection of oncovex^GM-CSF^ into melanoma lesions yielded an objective response rate of 28% in phase II clinical trials. Phase III clinical trials are ongoing [62].

However, oncolytic viruses all have the peculiarity of parental viruses and have some defects. Although HSV-1 is transmitted between cells and does not cause viremia, the most effective method for oncolytic HSV-1 is intracellular administration, which may not be suitable for intravenous infusion [105]. Because there are some drawbacks in intravenous administration, circulating antibodies may reduce the efficacy [106]. Viremia naturally causes viruses to be easily neutralized by antibodies; so the antineoplastic effect of intravenous administration of such viruses is limited in patients who have been treated or vaccinated. Clinical trials using oncolytic measles virus in the treatment of multiple myeloma fully demonstrated the adverse effects of circulating antibodies [107]. In dose-increasing studies, intravenous measles virus injection showed efficacy only when the dose reached a high dose of 10^11^ TCID50. In mice with transplanted tumors, intravenous injection of reovirus (REV) for 3 weeks after initial inhibition of tumor growth resulted in tumor regeneration, while the serum titer of anti- REVs antibodies increased [108]. Phase I study showed that 12 of 33 patients (36%) reached the maximum neutralizing REV antibody titer on the 7th day and 20 patients (61%) reached the maximum neutralizing REV antibody titer on the 14th day [109]. Hence, in the first week of treatment, speediness, repetitive, high-dose administration should be given before serum neutralizing antibodies rise, and should be combined with other anticancer therapies [106].

### Increase the efficacy of oHSV deliver to tumor cells

At present, in the application of ohsv, there are some problems that limit its therapeutic effect, whether intratumoral or intravenous injection, there are some defects. Intratumoral injection can ensure that virus particles reach the lesion directly, but it is difficult to spread to the lesion area outside the injection area. Intravenous injection provides an opportunity for the virus to infect all cancer cells, and is particularly effective in the treatment of metastatic lesions [110]. Nevertheless, viral particles injected into veins are bound to suffer innate immune responses from the host [111], which may result in the virus particles being neutralized by antibodies before reaching the target cells.

The receptors that bind oHSV to cells are repositioned to ensure that the virus is more readily accessible to cancer cells. The co-injection of oHSV and collagenase can degrade the extracellular matrix of tumors by collagenase, and make the region outside the injection site of virus particles diffuse. The method for delivery of the oHSV can be enhanced or reduced by pretreatment with antiangiogenesis molecules. When oHSV was administered by direct injection, prior injection of cyclic RGD peptide, an antiangiogenic agent, reduced tumor vascular permeability and infiltration of leukocytes [112]. When oHSV is injected intravenously, the blood-brain barrier will increase the difficulty of injection. In order to solve this problem, it has been proved that destruction of the blood-brain barrier through hypertonic solution of mannitol can increase the number of viruses reaching tumors [110]. Ultrasound technique is also used to enhance the permeability of cell membrane and the efficacy of chemotherapeutic drugs anti-cancer [113]. Shintani et al. showed that effective use of ultrasound technology to help oHSV-1 enter squamous cell carcinomas [114]. Combination with key immunoregulatory inhibitors can improve the efficacy of oncolytic virus. For example, a study showed that intravenous injection of anti-PD-1 antibodies combined with Reolysin was significantly more effective in treating subcutaneous melanoma in mice than intravenous injection of Reolysin or anti-PD-1 alone [115]. Combination of anti-PD-1 antibody therapy can improve NK cells’ effective lysis of REV infected malignant cells by reducing the activity of regulatory T cells. Phase I study of combined therapy of oncolytic virus T-Vec and pembrolizumab (anti-PD-1) for head and neck cancer has been completed [105].

Besides, the oHSV combination with chemotherapy is also an effective strategy for tumor treatment. Toyoizumi et al. showed that combining the HSV1716 with chemotherapeutic drug MMC to treat the human non-small cell lung cancer yielded profound efficacy [116]. This study showed that chemotherapy and oHSV can work together treat cancer, and this synergistic effect will strengthen anti-tumor ability. Co-administrated with cyclophosphamide, the anticancer activity of HrR3 improved effectively [117]. Cyclophosphamide can enhance the replication of oncolytic virus by inhibiting the immune response of the system and has better anti-cancer effect [118]. However, in all oncolytic theatmets, the long range of side effects of inducing via body anti-tumor immunity, such as the emergence of AIDs, requires close study.

## Conclusion

Oncolytic therapy, successes or failure hangs on the interaction of antiviral and antitumor immune responses between virus and host. HSV has been shown to be a site virus gets for oncolytic treatment because it is susceptible to genetic changes, deletions or mutations in genes with immunoregulatory function like ICP0, ICP 34.5, ICP 27, Us3 and UL39. This genetic alteration may result in an enhanced innate immune response, weakening viral replication and spreading in tumors.

At present, oHSV applied in clinical trials have not experienced serious adverse result and has achieved some effectiveness. For example, HSV1716 has been used for the treatment of oral squamous cell carcinoma and gloma [85–87]; G47Δ showed efficacy in glioma, breast cancer [93], malignant peripheral nerve sheath tumor [92], schwannoma [89], nasopharyngeal carcinoma [71], hepatocellular carcinoma [88], prostate cancer [87, 90, 91], colorectal cancer and thyroid carcinoma; NV1020 can effectively control liver metastasis and prolong survival via re-sensitizing to chemotherapy [100].

Although the deleted or mutated genes confer safety and selectivity to oHSV in the treatments of tumor cells, efficacy has been attenuated. Direct injection of oHSVs is usually preferred during treatment, but this procedure limits the delivery to the sites where the tumor actually occurs. Physical factors like the extracellular matrix can limit the initial distribution and external diffusion of oHSV in the tumors [119]. The inborn and acquired anti-virus immunity can limit the replication and spread of oHSV [120]. In oncolytic virotherapy, these are only some examples of the many hurdles to be overcome. This made it necessary to combine oHSV with other therapies. The expectations are to develop a combination therapy regimen that produces synergic action against tumor cells without overlapping side effects. For examples, the combinations of oHSV with collagenase can degrade the extracellular matrix of tumors by collagenase, and make the region outside the injection site of virus particles diffuse; Injection of circulating RGD peptide before oHSV infection can reduce the permeability of blood vessels and infiltration of leukocytes in tumors [112]; and many published combination joint research tested the efficacy of oHSV combined with immunotherapies and chemotherapies in vitro. These identified combinations have achieved some good results.

With the development of preclinical research into clinical application, it is more likely to achieve greater success in understanding the combination of oHSV and other treatments. In a word, there are many areas to be researched in development of oHSV combined with other therapies. But hopefully all would join hand to cure cancer patients.

## Abbreviations

APC: antigen presenting cell
DC: Dendritic cell
DNAM1: DNAX accessory molecule 1
EIF2a: eukaryotic Initiation Factor 2
GM-CSF: granulocyte macrophage colony-stimulating factor
IFN: interferon
oHSV: oncolytic herpes simplex virus
OPTN: optineurin
PKR: protein kinase R
RR: ribonucleotide reductase
TAP: transporter associated with antigen presentation
TK: thymidine kinase
TL: T lymphocyte
TNF-α: tumor necrosis factor
Vhs: virion host shutoff protein
